# Early Evolution and Historical Biogeography of Fishflies (Megaloptera: Chauliodinae): Implications from a Phylogeny Combining Fossil and Extant Taxa

**DOI:** 10.1371/journal.pone.0040345

**Published:** 2012-07-06

**Authors:** Xingyue Liu, Yongjie Wang, Chungkun Shih, Dong Ren, Ding Yang

**Affiliations:** 1 Department of Entomology, China Agricultural University, Beijing, People’s Republic of China; 2 College of Life Sciences, Capital Normal University, Beijing, People’s Republic of China; Field Museum of Natural History, United States of America

## Abstract

Fishflies (Corydalidae: Chauliodinae) are one of the main groups of the basal holometabolous insect order Megaloptera, with ca. 130 species distributed worldwide. A number of genera from the Southern Hemisphere show remarkably disjunctive distributions and are considered to be the austral remnants or “living fossils” of Gondwana. Hitherto, the evolutionary history of fishflies remains largely unexplored due to limited fossil record and incomplete knowledge of phylogenetic relationships. Here we describe two significant fossil species of fishflies, namely *Eochauliodes striolatus* gen. et sp. nov. and *Jurochauliodes ponomarenkoi* Wang & Zhang, 2010 (original designation for fossil larvae only), from the Middle Jurassic of Inner Mongolia, China. These fossils represent the earliest fishfly adults. Furthermore, we reconstruct the first phylogenetic hypothesis including all fossil and extant genera worldwide. Three main clades within Chauliodinae are recognized, i.e. the *Dysmicohermes* clade, the *Protochauliodes* clade, and the *Archichauliodes* clade. The phylogenetic and dispersal-vicariance (DIVA) analyses suggest Pangaean origin and global distribution of fishflies before the Middle Jurassic. The generic diversification of fishflies might have happened before the initial split of Pangaea, while some Gondwanan-originated clades were likely to be affected by the sequential breakup of Pangaea. The modern fauna of Asian fishflies were probably derived from their Gondwanan ancestor but not the direct descendents of the Mesozoic genera in Asia.

## Introduction

Fishflies are a group of little known insects in the subfamily Chauliodinae, family Corydalidae of Megaloptera, which is a basal holometabolous insect order and comprises only two extant families: Corydalidae and Sialidae, with larvae exclusively predacious and aquatic [1], [2]. The phylogeny and early evolution of Megaloptera attract great interest due to its old origin and significant systematic status in Holometabola [3], [4]. Traditionally, Megaloptera is considered to be monophyletic group, which is closely related to Raphidioptera, and Chauliodinae is the sister group of the subfamily Corydalinae [4]. Due to the difficulties to find morphological apomorphies, some controversial hypotheses on the monophyly and higher phylogeny of Megaloptera exist for a long period. For instance, Sialidae was mentioned to be the sister group of Raphidioptera based on the similar proximal fusion of M and CuA veins in the forewing and the same specialized telotrophic ovarioles [5]–[7], while a molecular phylogeny of Neuropterida assigned Corydalidae to be the sister group of Raphidioptera [3]. Nevertheless, the phylogenetic status of Megaloptera as the sister group of Neuroptera, as well as the monophyly of Megaloptera and Corydalidae is becoming stable through recent phylogenetic studies based on both morphological and multiple genes or mitochondrial genome data [8]–[11].

In the world, 128 species in 17 genera of extant fishflies are known, occupying about one third of the world megalopteran fauna, with most species well described in recent revisions [12]–[29]. Their species diversity is richest in Asia, especially the Oriental realm, with ca. 70 valid species (55% of world species) in five genera. Another region with high species diversity is the coastal area of eastern Australia and New Zealand where 25 endemic species in three genera are distributed [17]. The New World and southernmost Africa, as presumed hot spots for the generic diversification, harbor 11 endemic genera (65% of world genera) [4].

The fishflies live mainly in the subtropical or warm temperate regions, however, some species can adapt to certain harsh habitats and range in a rather broad area. For example, *Neochauliodes rotundatus* (Tjeder) can be found in cold temperate area of northernmost China or in some localities close to the metropolitan districts of Beijing with polluted aquatic habitats, while *Neochauliodes formosanus* (Okamoto) has the widest distribution among all Asian Megaloptera species ranging from northern Vietnam through major part of mainland China to the islands of Tsushima, Taiwan, and Hainan, and even in high-elevation regions of the Qinghai-Tibet Plateau [4]. Nevertheless, most fishfly species are narrowly distributed and rarely found in the wild, due to their weak dispersal capacity [30], [31].

Considering the global geographical distribution, extant fishflies occur in all zoogeographical realms, but, show a remarkably discontinuous distribution due to their absence in the western Palaearctic realm and most parts of the Afrotropical and Neotropical realms. Some morphologically similar genera, e.g. *Protochauliodes* (a few species also occur in western North America), *Taeniochauliodes*, and *Nothochauliodes*, are distributed in Australia, South Africa, and Chile, respectively seemingly representing the austral relicts after the Mesozoic continental drift. Disjunctive distributions can be also found in *Archichauliodes* occurring in eastern Australia, New Zealand, and Chile.

Historically, fishflies might have originated no later than the Middle Jurassic based on fossil evidence and their current austral distribution of some relict genera, which can be considered as “living fossils” [22], [32]. A time-scale for the diversification of Neuropterida indicates that the Corydalidae might originate between the Early Permian and Early Jurassic (251 MYA on average), while the generic diversification within Corydalidae might have happened during the Early Triassic to Early Cretaceous (180 MYA on average) [3]. The oldest fossil record of Sialidae, which is the conventional sister group of Corydalidae, from the Early Jurassic of Germany (185 MYA) is another circumstantial evidence for the origin of Corydalidae no later than the Early Jurassic [33]. Nevertheless, definite fossil of Corydalidae has never been found before the Middle Jurassic. Despite this commonly accepted perspective, the evolutionary history of fishflies remains largely unexplored due to the limited fossil record. Currently, the Corydalidae fossils are extremely rare, including one species (*Jurochauliodes ponomarenkoi* Wang & Zhang) with only larvae described from the Middle Jurassic, one species (*Cretochaulus lacustris* Ponomarenko) with both adults and larvae described from the Early Cretaceous, one species (*Chauliosialis sukatshevae* Ponomarenko) with only first instar larva described from the Late Cretaceous, and two species (*Chauliodes carsteni* Wichard and *Chauliodes prisca* Pictet) from Eocene Baltic amber [32], [34]–[36]. Among these fossils, five species belong to the subfamily Chauliodinae, however the adults of extinct fishflies are poorly known. Thus, the insufficient morphological data from scarce fossil material apparently restricts the inference of the phylogeny including both extinct and extant fishfly genera. Moreover, the previous phylogenetic analysis of Chauliodinae is also incomplete [22] due to the absence of several austral endemic genera from Africa, Australia, and South America as well as two enigmatic genera from western North America, which resulted in limited understanding of the evolutionary trend of this archaic insect group.

Here we report a new fishfly genus and species (*Eochauliodes striolatus* gen. et sp. nov.) and the first adult of *Jurochauliodes ponomarenkoi* Wang & Zhang from the Middle Jurassic (Bathonian-Callovian) of the Jiulongshan Formation in Daohugou (41°18′30″N, 119°13′00″E) of Ningcheng County, Inner Mongolia, China. These fossils represent the historically earliest fishfly adults. Based on these invaluable fossils, the first phylogeny including all extinct and extant fishfly genera is reconstructed. Under the current phylogenetic framework, this paper sheds new light on the early evolution and historical biogeography of fishflies.

## Results

### Systematic Paleontology

Class Insecta Linnaeus, 1758; Order Megaloptera Latreille, 1803; Family Corydalidae Leach, 1815; Subfamily Chauliodinae van der Weele, 1909.

**Figure 1:**
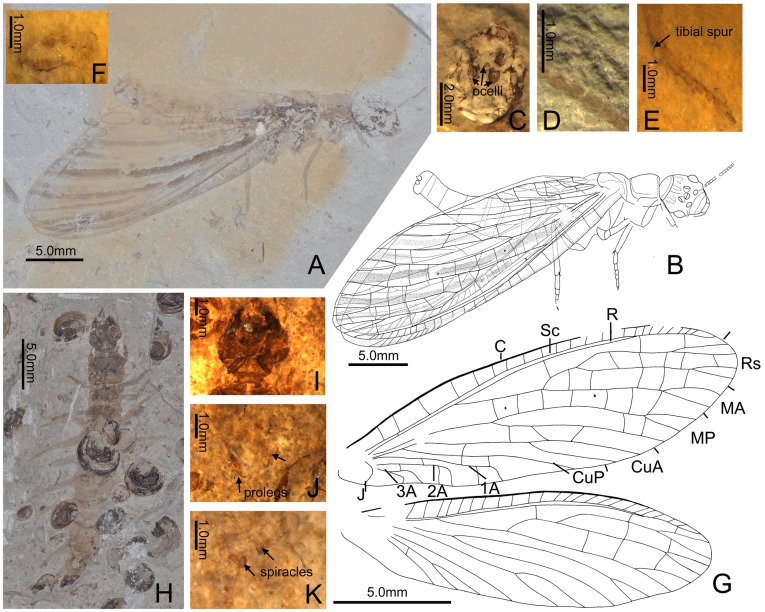
*Eochauliodes striolatus* gen. et sp. nov. A–G, Adult, holotype CNU-MEG-NN2011004 P, part: A, Habitus; B, Habitus illustration; C, Head, showing ocellar triangle; D, Antenna; E, Apex of mid leg, showing tibial spur and hairs; F, Genitalia; G, Venation of forewing and hindwing; H–K, Larva, CNU-MEG-NN2011008: H, Habitus; I, Head; J, Abdominal apex, showing prolegs; K, Abdominal apex, showing enlarged spiracles on tergite 8. Abbreviations: C: costa, Sc: subcosta, R: radius, Rs: radial sector, MA: anterior media, MP: posterior media, CuA: anterior cubitus, CuP: posterior cubitus, A: anal vein, J: jugal vein.

Genus ***Eochauliodes*** **gen. nov.** (Fig. 1).

urn:lsid:zoobank.org:act:80BFDAB1-1042-42F4-B1FC-C9A1DBEABDF3.

#### Type species

Eochauliodes striolatus sp. nov.

#### Etymology

The generic name is a combination of *eo* (Greek, meaning beginning) and *chauliodes* (Greek, meaning possessing prominent teeth, frequently used as the etyma of the genus name of fishflies).

#### Diagnosis

Adult medium-sized (forewing length ∼30.0 mm). Antenna (Fig. 1D) filiform. Ocelli (Fig. 1C) close to each other. Wings (Fig. 1G) narrowly elongated, nearly 3.0 times as long as wide; membrane hyaline, with a few dark stripes along longitudinal veins. Nygmata (Fig. 1G) present between MA and MP. Rs 2-branched, each branch with a distal fork; MA bifurcated distad; MP 2-branched, with anterior branch distally forked; 1A, 2A, and 3A all present in forewing, each with two branches and curved near wing margin; 1A and 2A connected by a short crossvein in forewing. Larva (Fig. 1H) medium-sized (body length of final instar larvae around 40.0 mm by estimation). Antennae with terminal two segments nearly as long as 2nd segment. Pronotum subquadrate, slightly wider than long. Lateral abdominal gills (Fig. 1H) on segments 1–5 distinctly longer than width of respective segments, and also longer than hind legs. Spiracles on segment 8 (Fig. 1K) not protruding as a pair of tubes.

***Eochauliodes striolatus sp. nov.*** (Fig. 1).

urn:lsid:zoobank.org:act:B07468A0-E2FA-4AFD-A291-ED756C46B11E.

#### Diagnosis

Wings with rather long stripes along longitudinal veins from base to apex. All branches of Rs and MA are bifurcated in both wings.

#### Description

Adult. Body length 28.2 mm; head width 4.0 mm; forewing length 28.7–31.5 mm and width 8.5–10.5 mm; hindwing length 26.3 mm and width 6.6 mm. Head (Fig. 1C) ovoid, vertex subtrapezoidal, distinctly rugose; clypeus subtriangular; occiput short, transversely elliptical; compound eyes elliptical and protruded; ocellar triangle present at middle of frons, ocellus ovoid with semicircular basal sclerite and close to each other; right antennae partially preserved, filiform. Prothorax (Fig. 1B) compressed from lateral side in holotype, subquadrate, slightly shorter than head; meso- and metathorax robust, much larger than prothorax. Legs (Fig. 1E) slender, densely setose; forelegs incompletely preserved; mid- and hindlegs well preserved, tibiae with a small tibial spur, tarsi simple, terminally with a small claw. All wings (Fig. 1A) preserved but overlapped; narrowly elliptical, nearly 3.0 times as long as wide; proximal half of costal area in forewing much broader than distal half; hindwing with feebly developed anal area; membrane with approximately five long dark stripes along C, Rs, MA, MP, and CuA, some stripes occasionally interrupted, but strip between MA and MP longest and always patterned continuously in examined specimens, additional short dark markings also present on distal margins; pterostigmatic area dark; two nygmata present at middle of forewing between MA and MP. Forewing venation (Fig. 1G): costal crossveins simple, numbers varying around 25, with distal ones weak; Sc and R fused distad; a rather short crossvein present between Sc and R proximally; three crossveins present between R and Rs; Rs originate from R, with two main branches, each branch straightly extended with bifurcated fork distad; MA proximally fused with Rs, distally with a bifurcated fork; MP bifurcated proximal to separation of MA from Rs, straight, anterior branch with bifurcated fork distad and posterior branch simple; Cu forked at wing base, CuA bifurcated medially into long distal fork, CuP simple; 1A with length almost equal to half of CuA, distally bifurcated and curved posteriorly; 2A with length almost equal to half of 1A, medially connected with 1A by short crossvein, distally bifurcated and curved posteriorly; 3A rather short and bifurcated from base and curved posteriorly; J present, simple. Hindwing venation (Fig. 1G): almost same as forewing venation, but base of MA absent or not preserved, 1A straight, most crossveins and anal veins not preserved. Abdomen with eight segments visible; caudal segment (Fig. 1F) with arched posterior margin and pair of small setose lobes, resembling ninth tergum and ectoprocts of extant fishflies.

#### Larva

Body length ∼39.0 mm; head width 4.5 mm; pronotum length 4.4 mm and width 5.2 mm (CNU-MEG-NN2011009). Body length ∼28.5 mm; head width 3.7 mm; pronotum length 3.3 mm and width 4.0 mm (CNU-MEG-NN2011008). Head and thorax coloration (Fig. 1H) conspicuously dark, and abdomen pale colored. Head (Fig. 1I) nearly as long as wide, lateral margins distinctly convex, forming subtrapezoidal vertex. Labrum subtriangular. Antenna with terminal two segments nearly as long as 2nd segment. Mandible much shorter than head (probably due to deformation during compression), with three rather small teeth on inner margins. Pronotum subquadrate, slightly wider than long. Meso- and metathorax slightly narrower than pronotum, and almost half as long as pronotum. Lateral abdominal gills (Fig. 1H) on segments 1–5 distinctly longer than width of respective segments, and also longer than hind legs. Spiracles on segment 8 (Fig. 1K) not protruding as pair of tubes.

#### Type material

Holotype, CNU-MEG-NN2011004 P/C (part and counterpart), specimen consists of a nearly complete adult, with four wings stretched out but mostly overlapping. Paratypes: CNU-MEG-NN2011005 P/C (part and counterpart), specimen consists of a deformed body with abdominal apex not preserved and with wings overlapped; CNU-MEG-NN2011006, specimen consists of only one forewing, which lacks the tip and the anal area; CNU-MEG-NN2011007 P/C (part and counterpart), specimen consists of only one forewing, which lacks the posterior marginal part.

#### Other material

CNU-MEG-NN2011008, specimen consists of a nearly complete larva; CNU-MEG-NN2011009 P/C (part and counterpart), specimen consists of a larva with abdomen mostly damaged; CNU-MEG-NN-2011010, specimen consists of a larva with only head and thorax preserved; CNU-MEG-NN-2011011, specimen consists of a nearly complete larva with head lost.

#### Type locality

Daohugou Village, Shantou Township, Ningcheng County, Inner Mongolia, China (41°18′30″N, 119°13′00″E).

#### Type horizon

Jiulongshan Formation, Bathonian-Callovian boundary, Middle Jurassic.

#### Etymology

The specific name is from the Latin word *striolatus* (meaning, bearing narrow markings), referring to the striking wing pattern of the new species.

#### Remarks

*Eochauliodes striolatus* gen. et sp. nov., representing a new fishfly species, is distinguished in appearance from all other species of Chauliodinae by wings with longitudinal dark stripes. Based on the venation, the new species seems to be similar to *Cretochaulus lacustris* Ponomarenko, 1976 from Early Cretaceous of Russia and the species of some extant genera (*Protochauliodes*, *Neohermes*, and *Madachauliodes*) by the Rs with both main branches bifurcated and the anterior branch of MP bifurcated in hindwing. However, it differs from all these genera and species by the bifurcated MA, and it can be also separated from *Protochauliodes* and *Neohermes* by the 2A connected to 1A by a short crossvein and the bifurcated anterior branch of MP in the forewing. The identification of the larvae of the new species is according to the similar capsule width between adults and large larvae. The larva of *E. striolatus* differs from that of *J. ponomarenkoi* by the following characters: head and thorax rather dark, pronotum slightly wider than long, and lateral abdominal gills distinctly longer than hind legs.

**Figure 2:**
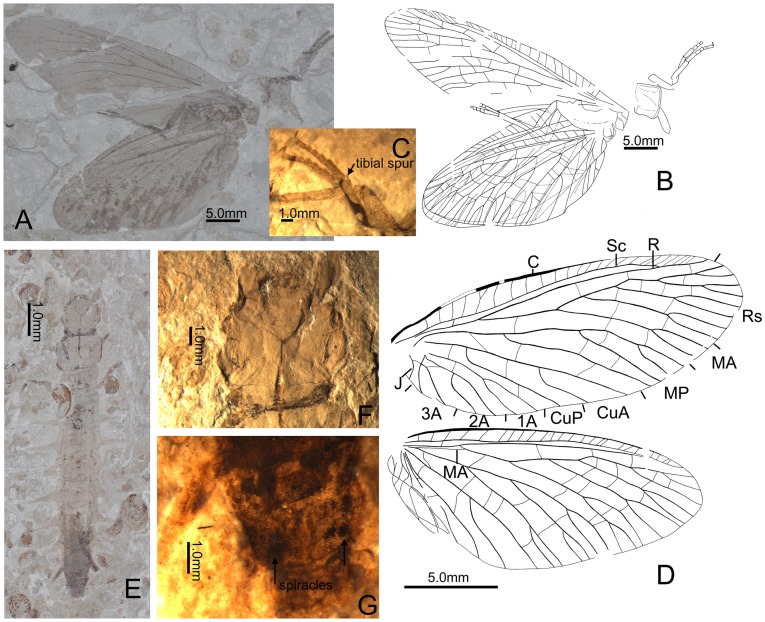
*Jurochauliodes ponomarenkoi* Wang & Zhang 2010. A–D, Adult, CNU-MEG-NN2011001 P, part: A, Habitus; B, Habitus illustration; C, Fore and mid legs, showing tibial spur and hairs; D, Venation of forewing and hindwing; E–G, Larva, CNU-MEG-NN2011003: E, Habitus; F, Head; G, Abdominal apex, showing enlarged spiracles on tergite 8.

Genus ***Jurochauliodes*** **Wang & Zhang, 2010** (Fig. 2).

*Jurochauliodes* Wang & Zhang, 2010∶777. Type species: *Jurochauliodes ponomarenkoi* Wang & Zhang, 2010∶777 (original designation for fossil larvae only).

#### Emended diagnosis

Adult medium-sized (forewing length ∼35.0 mm). Wings (Figs. 2B, D) broadly elliptical, nearly 2.5 times as long as wide; membrane hyaline, slightly dark on forewing, but without distinct pigmented markings. Rs 4-branched, each branch bifurcated; MA bifurcated distad; MP 2-branched, with anterior branch distally 3 or 4-branched, and with posterior branch usually bifurcated; 1A, 2A, and 3A all present in forewing, each with two branches and straightly extending; 1A and 2A connected by a short crossvein in forewing. Larva (Fig. 2E) medium-sized (body length of final instar larva about 50.0 mm by estimation). Pronotum subquadrate, distinctly wider than long, and also wider than head. Lateral abdominal gills on segments nearly as long as width of respective segments, and slightly shorter than hind legs. Spiracles on segment 8 not protruding as a pair of tubes.

***Jurochauliodes ponomarenkoi*** **Wang & Zhang, 2010** (Fig. 2).

*Jurochauliodes ponomarenkoi* Wang & Zhang, 2010∶777.

#### Type locality

Daohugou Village, Shantou Township, Ningcheng County, Inner Mongolia, China (41°18′30″N, 119°13′00″E).

#### Type horizon

Jiulongshan Formation, Bathonian-Callovian boundary, Middle Jurassic.

#### Emended diagnosis

Wings broad; Rs with four main branches, all of which consist of a bifurcated fork; MP with two main branches, and anterior branch 3 or 4-branched.

#### Description

Adult. Prothorax length 5.1 mm and width 5.3 mm; forewing length 34.6/39.5 mm and width 14.4 mm; hindwing length 30.0 mm and width 13.3 mm. Head not preserved. Prothorax (Fig. 2B) subquadrate; meso- and metathorax not preserved. Legs (Fig. 2C) slender, densely setose; tibiae with a small tibial spur, tarsi simple, terminally with a small claw. All wings (Fig. 2A) preserved but one forewing and two hindwings overlapped; broadly elliptical, nearly 2.0 times as long as wide; proximal half of costal area in forewing much broader than distal half; hindwing with well developed anal area, which seems twisted during compression; membrane hyaline, with forewings slightly darkened and bearing a few pale stripes at middle; pterostigmatic area indistinct; nygmata absent but probably due to poor preservation. Forewing venation (Figs. 2B, D): costal crossveins mostly simple except for one anteriorly bifurcated, numbers varying around 30, with distal ones weak; Sc and R fused distad; a rather short crossvein present between Sc and R proximally; three crossveins present between R and Rs; Rs originate from R, with four main branches, each branch straightly extending with a bifurcated fork at middle; MA proximally fused with Rs, with a bifurcated fork at distal 1/3; MP bifurcated proximal to the separation of MA from Rs, straightly extending, anterior branch bifurcated into one forked and one simple subbranch, and posterior branch bifurcated at distal 1/3 or simple; Cu forked at proximal 1/5, CuA bifurcated medially into long fork, CuP simple with base arcuately curved; 1A with length almost equal to half of CuA, straight and bifurcated at middle; 2A slightly shorter than 1A, medially connected with 1A by short crossvein, bifurcated at middle and slightly sinuate; 3A nearly 1/2 as long as 2A, bifurcated from base and slightly sinuate; J present, simple. Hindwing venation (Fig. 2D): almost same with forewing venation, but base of MA present as weak oblique vein connected to MP at base, MA distally trifurcated, MP with anterior branch bearing two bifurcated subbranches. Abdomen not preserved.

#### Larva

See description by Wang & Zhang [32].

#### Material examined

CNU-MEG-NN2011001 P/C (part and counterpart), specimen consists of well preserved adult with wings, legs, and prothorax, with three wings overlapped; CNU-MEG-NN2011002 P/C (part and counterpart), specimen consists of a well preserved larva; CNU-MEG-NN-2011003, specimen consists of a well preserved larva; CNU-MEG-NN-2011012, specimen consists of a nearly complete larva with head partly damaged.

#### Remarks

The adult fossil described here as *J. ponomarenkoi* distinctly differs from all known Corydalidae species based on combination of the following morphological features: all branches of Rs and MA bifurcated, and MP with both main branches bearing additional subbranches. However, the bifurcated posterior branch of Rs shows that this adult should belong to Chauliodinae. The variation of the branching of MA and MP is present as that the MA is distally bifurcated in both forewings but trifurcated in a hindwing, the anterior branch of MP is distally 3-branched in forewing but 4-branched in hindwing, and the posterior branch of MP is bifurcated in one forewing but simple in another forewing. The genus *Jurochauliodes* was established by Wang and Zhang based on only fossil larvae [32], whose generic status is difficult to be clarified because the fishfly larva does not contain sufficient morphological diagnosis for distinguishing all the genera. Although the larva described by Wang and Zhang [32] differs from the other fossil larvae of fishflies by the larger body size and the short lateral abdominal gills, it seems quite similar to the extant genus *Dysmicohermes* from the western Nearctic realm [15] and hard to be distinguished from *Dysmicohermes* without regard to the pronotum wider than head. Fortunately, the present discovery links this relatively large fossil adult with the *Jurochauliodes* larvae, which also have larger body size. Moreover, the morphological similarity, such as the bifurcation of all Rs branches, MA, and MP branches in the hindwing, between *Jurochauliodes* and *Dysmicohermes* also support our identification.

### Phylogenetic Analysis

**Figure 3:**
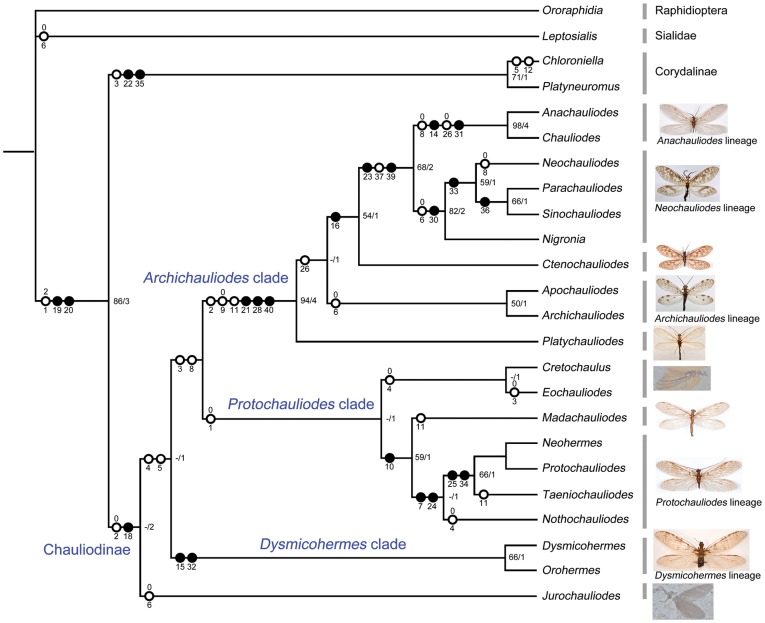
Parsimonious phylogeny of world fishfly genera. Bootstrap values/Bremer’s decay indices are indicated at each node, unambiguous apomorphies were mapped. Black circle represents non-homoplastic change, white circle represents homoplastic change, characters states for multistate characters and reversals are placed above corresponding circles. Habitus photos of representative genera of all lineages shown at right.

The heuristic analysis resulted in two most parsimonious (MP) trees (length = 69, consistency index (CI) = 0.6087, retention index (RI) = 0.8500). One MP tree has a fully bifurcated topology (Fig. 3), while the other one, which is identical to the strict consensus tree (Fig. S1), has mostly consistent topology, only leaving the monophyletic group composed of *Taeniochauliodes*, *Protochauliodes*, and *Neohermes* to be unresolved. The former intergeneric phylogeny of fishflies was made by an incomplete sampling due to lack of a number of significant taxa (all Afrotropical and some Nearctic/Neotropical genera) [22], while the present phylogeny includes all extant and fossil genera.

Here, the subfamily Chauliodinae is monophyletic based on the bifurcated posterior branch of Rs in the forewing and the specialized eighth abdominal spiracles in the larvae. The Middle Jurassic genus *Jurochauliodes*, with basal connection between 1A and 2A at stem of 2A in the forewing as the autapomorphic character, was assigned to be the sister of the remaining fishfly genera, which were divided into three main clades, i.e. the *Dysmicohermes* clade (*Dysmicohermes* and *Orohermes*), the *Protochauliodes* clade (*Cretochaulus*, *Eochauliodes*, *Madachauliodes*, and the *Protochauliodes* lineage), and the *Archichauliodes* clade (*Platychauliodes*, the *Archichauliodes* lineage, the *Anachauliodes* lineage, *Ctenochauliodes*, and the *Neochauliodes* lineage). The other Middle Jurassic genus *Eochauliodes* possesses bifurcated MA in the forewing as its autapomorphic character. It is notable that the *Eochauliodes* and Early Cretaceous *Cretochaulus* are sister group and clustered with *Madachauliodes* + the *Protochauliodes* lineage based on the bifurcated anterior branch of Rs. Compared with the previous phylogeny of fishflies [22], the major difference at present refers to the phylogenetic status of the genera from the Southern Hemisphere and the western North America. These genera, which were assumed to form a monophyletic clade [4], [22], are heterogeneous and respectively placed into the three main clades recognized above. Nevertheless, the monophyly of the *Protochauliodes* lineage was corroborated based on the unique fusion between stem of 1A and anterior branch of 2A in the forewing. Furthermore, all Asian genera plus eastern North American *Chauliodes* and *Nigronia* were recovered as same as the “clade 1” shown in the previous phylogeny [22] and its monophyly was supported by the pectinate or subserrate antennae. The placement of *Ctenochauliodes* and the monophyly of the *Anachauliodes* and *Neochauliodes* lineages are also consistent with the former phylogeny, but *Nigronia* was assigned to be the sister of *Neochauliodes* + *Parachauliodes* lineage.

## Discussion

### Implications of Unique Morphological Traits in Mesozoic Fishflies

The rather similar larval morphology between the Mesozoic and modern fishflies suggest that the larvae have evolved the aquatic and predatory habits in the Jurassic. The larvae of extant fishflies live in various habitats such as streams, rivers, swamps, and ponds, but have been never reported to be found in lakes [15], because they require rich dissolved oxygen for respiration. However, Ponomarenko mentioned that the Early Cretaceous fishfly genus *Cretochaulus* inhabited in a large lake [34]. The palaeoenvironment of Jiulongshan Formation, the locality of Middle Jurassic fishflies described above, was considered as shallow lacustrine environment near shore [37], which is similar to that of *Cretochaulus*. Based on the above-mentioned larval morphology, i.e. lateral abdominal gills and spiracles, the larvae of Mesozoic fishflies should also prefer water habitats with rich dissolved oxygen. During the Jurassic, oxygen content on earth (∼26%) was much higher than present (20–21%) [38], therefore, the near shore area of a lake might have high oxygen content in water, which was suitable for the Mesozoic fishfly larvae.

The striking wing marking pattern of *Eochauliodes* is probably a unique autapomorphic character of this group. This pattern of markings might be a camouflage, judging from the alternately dark and pale stripes, which are also present in Mesozoic Orthoptera, Hemiptera, and Neuroptera etc. [39]. Similar black and white wing patterns are also observed in some extant diurnal fishfly and dobsonfly species, such as *Nigronia* and *Neurhermes*, which are considered to imitate some poisonous moths [40]. However, we ruled out the possibility that this pattern of *Eochauliodes* represents aposematic coloration, because coeval large and poisonous moths had not yet evolved during the Middle Jurassic [41].

### Pangaean Distribution of Fishflies

Hitherto, all Paleozoic and Mesozoic Megaloptera were found in the Northern Hemisphere, especially in Eurasia [42], except for Euchauliodidae, which is a putative family placed in Megaloptera from the Triassic of South Africa [43]. The present DIVA analysis locates the ancestral distribution for all Mesozoic and extant fishflies in Asia-western North America-southern African continent or Asia-western North America-southern South America, which apparently indicates that the global Pangaean distribution of fishflies crossing Northern and Southern Hemisphere might have been formed before the Middle Jurassic. Furthermore, the diversification of fishflies had already initiated during this period because two Middle Jurassic genera and the extant *Dysmicohermes* clade from western North America arose. However, no significant plate drift, such as the split of Pangaea, can be found to account for these early vicariant events, and therefore some orogenic events or climate change might play an important role in shaping the early fauna of fishflies. It is worth to mention the vicariance between the ancestral *Dysmicohermes* clade from the Northern Hemisphere and the common ancestor of the *Protochauliodes* and *Archichauliodes* clades from the Southern Hemisphere. The fishflies preferably live in the subtropical and warm temperate areas [1]. In Early Jurassic there were extensive tropical summerwet and desert regions, which are unfavorable for fishflies, between two warm temperate regions respectively in Northern and Southern Hemispheres [41]. Therefore, the equatorial zone might present a barrier for this vicariance event. Subsequently in the Middle Jurassic, the unsuitably dry areas for fishflies were reduced in the equatorial zone [41] and some pathways might be formed again for the faunal exchange between Northern and Southern Hemispheres, for instance, enabling the global dispersal for the ancestral *Protochauliodes* clade.

### Breakup of Pangaea and Diversification of Fishflies

The diversification of various flora and fauna during Mesozoic and Early Cenozoic is known to be correlated with the breakup of Pangaea [41]. The initial split of Pangaea began in the Early-Middle Jurassic (∼175 MYA), ultimately giving rise to the supercontinents Laurasia and Gondwana [41]. Considering the major landmasses of Laurasia, North America was connected with Europe but widely separated from Asia from the Early to Late Jurassic [44]. Subsequently, two palaeocontinents, Euramerica (Europe and eastern North America) and Asiamerica (Asia and western North America), were formed and persisted until the end of Cretaceous [44]. Considering the major landmasses of Gondwana, the rifting of Gondwana began in the Early-Middle Jurassic (∼180 MYA), starting with that between eastern Gondwana (Africa, India, Madagascar) and western Gondwana (all other southern landmasses) [41], [45]. Madagascar and India broke away from Africa and began moving southeast in the Early Cretaceous (121 MYA) [46], while India separated from Madagascar in the Late Cretaceous (88–84 MYA), drifting northward, eventually to collide with Asia in the Early Tertiary (∼50 MYA) [45], [47]. South America began to separate from Africa in the Early Cretaceous (135 MYA), and southern South America drifted southwest and joined Antarctica together with New Zealand and Australia into a landmass, which was completely separated among each other in Oligocene (30–28 MYA) [45], [48].

Besides the classic study on the basal subfamilies of Chironomidae by Brundin [49], the evolution of many living families of insects originated in the Mesozoic were reported to be clearly shaped by the breakup of Pangaea, Laurasia, and Gondwana, which is of greatest interest and a premier example of how geological events led to repeated and widespread vicariance, or separation, of closely related lineages [41]. Comprehensive analyses aiming to reconstruct a general biogeographical pattern among main landmasses were made by Sanmartín et al. and Sanmartín and Roquist [44], [45]. For instance, a southern Gondwana pattern (SGP), i.e. (Africa + (New Zealand + (Southern South America + Australia))), was proposed for animals by Sanmartín and Ronquist [45]. However, recent molecular phylogenetic studies have challenged the traditional time table of the sequential continental breakup and indicated that the connection among major landmasses might be prolonged until the Late Cretaceous based on the timing of divergence [50]. Moreover, inconsistent hypotheses also frequently came out for certain animal or plant group [51].

The present ancestral distribution reconstruction strongly suggests the Gondwanan origin of most fishfly genera except *Jurochauliodes* and the *Dysmicohermes* clade, and the diversification of these genera is likely to be affected by the sequential breakup of Pangaea. The *Protochauliodes* clade includes four morphologically similar extant genera from southern South America, eastern Australia, Madagascar, and South Africa. The phylogenetic relationships among these genera generally conform to the separation between Madagascar and Africa + South America [41], [45], while the distribution of *Protochauliodes* in both Australia and South America may reflect the trans-Antarctic dispersal of its ancestor [45]. The DIVA analysis shows that the ancestral distribution of the monophyletic group including *Madachauliodes* + the *Protochauliodes* lineage might be either restricted to the Gondwana or include the western North America together with some Gondwanan landmasses, however the involvement the western North America is apparently due to the North American distribution of the sister pair *Neohermes* and *Protochauliodes*, which are embedded into the other austral endemic genera. Penny stated that the ancestral *Protochauliodes* might have originated from Gondwana and dispersed northward to western North America, which was considered to be a secondary origin centre for fishflies [30]. Thus, *Neohermes* and the North American endemic species group of *Protochauliodes* might be separated when their common ancestor arrived at North America, however it will make *Protochauliodes* to be paraphyletic, which needs further clarification. If this hypothesis is true, the western North America would be excluded from the ancestral distribution of the subclade of *Madachauliodes* + the *Protochauliodes* lineage, and the Mesozoic *Eochauliodes* + *Cretochaulus* from Asia might be isolated with the former Gondwanan-originated fishfles due to the initial split between Laurasia and Gondwana.

Similarly, considering the *Archichauliodes* clade, multiple ancestral distributions were reconstructed by DIVA analysis and some of them also include Asia plus certain Gondwanan areas, whereas the others are combinations of only Gondwanan areas. As fossil evidence indicates that the genus *Chauliodes*, a young taxon within the *Archichauliodes* clade, originated no later than Eocene, the preceding divergence might be underway during Mesozoic and early Cenozoic when Southern Africa was isolated from Asia after the initial breakup of Pangaea. Therefore, if the common ancestor of this clade occurred in both Asia and southern Africa, its origin time should fall in the Early Jurassic or even earlier when these two areas were still connected. Alternatively, if the ancestral distribution of the *Archichauliodes* clade is confined within Gondwana, the diversification of this clade could also initiate somewhat later after the split between Laurasia and Gondwana, and a dispersal event from Gondwana to Asia should be required to account for the ancestral distribution of all extant Asian fishflies plus eastern North American *Chauliodes* and *Nigronia*. The present phylogenetic pattern within the *Archichauliodes* clade does not support similar deep vicariance between Laurasia and Gondwana as the basal divergence within the *Protochauliodes* clade, but seemingly conform to the northern Gondwana pattern, i.e. Africa + (Asia + Australia), proposed by Sanmartín and Ronquist [45], which suggests that a Gondwanan origin of the *Archichauliodes* clade is preferable.

### Fall and Rise of the Asian Fishflies

Penny pointed out that the Asian dobsonfly genera could have been present either in South Asia from a previous vicariant event (perhaps the sundering of Pangaea) or been introduced as a single or multiple ancestors on the Gondwanan-derived Indian subcontinent as it drifted northward, and the historical biogeography of fishflies may also fit this hypothesis [30]. Based on the fossil record of fishfies from the Early Cretaceous [34], Liu and Yang rejected the assumption that the extant Asian fishflies were brought by India from Gondwana and considered that they might have originated before the splitting of Laurasia and Gondwana [32], in other words, being the direct descendants of the Mesozoic fishflies from Asia.

In the present phylogeny, it is notable that all Mesozoic fishflies from Asia occupy relatively basal positions, while the extant Asian fishflies appear to be separated from some austral endemic genera. As discussed above, all extant Asian fishflies plus *Chauliodes* and *Nigronia* from eastern North America, which comprise the *Neochauliodes* subclade, might preferably arise from certain Gondwana-originated ancestor. Thus, how did their ancestor colonize the Northern Hemisphere across the deep ocean from Southern Hemisphere?

The records of the genus *Chauliodes* and an undescribed species closely related to *Neochauliodes* from the Eocene Baltic amber [35], [36], [52] indicate that their ancestor had been present in Eurasia no later than Eocene. Before or during that period, two continental connections were mentioned to exist as the pathways for the animal dispersal from Southern to Northern Hemisphere, i.e. the insular connection between South America and western North America and the collision between Indian subcontinent and Eurasia, whereas the former route is considered to be less significant for the faunal exchange [53].

If the ancestor of the *Neochauliodes* subclade made the dispersal from certain Gondwanan landmass to western North America via the link as a pattern of islands, it could have moved to Asia from western North America via the Beringian Bridge or by crossing the mid-continental area of North America (formed by closure of the mid-continental seaway at the end of Cretaceous), the Thulean Bridge (an important land bridge for trans-Atlantic dispersal), and the Turgai Strait (a significant barrier between Europe and Asia) through eastern North America and Europe [44]. The latter dispersal requires long-distance migration and crossing broad geographic barriers, thus seems rather difficult to take place. The dispersal via Beringian Bridge in the Early Tertiary for the subtropical or warm temperate fauna and flora, although favored by many authors [54], [55], was considered to be less likely than the trans-Atlantic dispersal based on comprehensive biogeographic analyses by Sanmartín *et al*. [44], which was supported by the palaeobotanical evidence indicating that the Eocene Beringian Bridge was primarily covered by deciduous hardwood forests, with only a thin southern fringe of evergreen, subtropical communities. Furthermore, the extant fauna of fishflies between western North America and Asia are considerably different, without close phylogenetic relationships. Therefore, the ancestor of the *Neochauliodes* subclade probably never occurred in western North America and managed the eastward migration to Asia.

By excluding the dispersal from America to Asia, the most possible route for the northward dispersal of the ancestor of *Neochauliodes* subclade could be the rafting of the Indian subcontinent from eastern Gondwana to Asia, which was also stated as one explanation for the origin of modern dobsonflies from Asia by Penny [30]. This dispersal event might have taken place in Eocene or even earlier in the latest Cretaceous as the faunal exchange was detected before the collision of these two plates [50], [53]. After the landing of the ancestors of the *Neochauliodes* subclade in Asia, it extensively diversified and probably expanded the distribution to Europe along the coasts and islands of the Tethys Seaway, which was mentioned to be a linkage of the flora between Asia and Europe in the Early Tertiary [56], [57], and ultimately reached eastern North America via the Thulean Bridge. However, the ancestor of the *Neochauliodes* subclade probably never managed the dispersal to western North America either by the Beringian Bridge or through the mid-continental area of North America, which is consistent with the absence of *Chauliodes* and *Nigronia* in western North America.

The absence of fishflies with close affinity of the Mesozoic relatives in Cenozoic Eurasia indicates that all Mesozoic fishflies in Asia might be extinct by the end of Cretaceous or the beginning of Tertiary. Although the effects of the K/T extinction on insects at the family level are considered to be imperceptible [41], some regional extinction of insects, such as the Raphidioptera from Southern Hemisphere and the Neuroptera family Kalligrammatidae from Eurasia, might have happened due to the K/T event [41], [58]. The decreased content of dissolved oxygen at that time probably played a key role in eliminating the Mesozoic Asian fishflies, whose larvae lacking abdominal spiracle tubes might strongly rely on the high content of dissolved oxygen in freshwater. The well developed larval spiracle tubes in extant fishflies from Asia as well as *Chauliodes* and *Nigronia* might be evolved for breathing the atmospheric oxygen, an adaptation of aquatic habitat with decreased dissolved oxygen, and enable them living successfully in Cenozoic Asia. Besides the reduced content of the dissolved oxygen, exceptionally cold climate in the Early Cretaceous of eastern Asia [59] may be another reason for the extinction of the Mesozoic fishflies from this area.

Consequently, the fishflies in Asia shows a distinct faunal turnover from Mesozoic to present that all Jurassic and Cretaceous genera faded away at the end of Mesozoic, while new genera rose after their colonization from Gondwana and finally formed the modern fauna from Asia – the most diversified one in the world.

### Conclusions

The present discovery of the Middle Jurassic fishflies, particularly the first report of the earliest fishfly adults, is of great significance to understand the early evolution and historical biogeography of the subfamily Chauliodinae. The first phylogenetic analysis and ancestral distribution reconstruction combining all extinct and extant fishfly genera confirm the origin of fishflies before the split of Pangaea. The result also suggests that the generic diversification of fishflies was shaped not only by the sequential Pangaean breakup but also by the paleoclimate change before the split of Pangaea. Finally, it demonstrates that the modern fauna of Asian fishflies are probably derived from their Gondwanan ancestors, but not the direct descendents of the Mesozoic genera currently known from Asia. To test the present result and further reveal the evolutionary history of fishflies, reconstruction of the phylogeny by adding the molecular data and a time-scale based on the divergence time estimation should be made.

**Figure 4:**
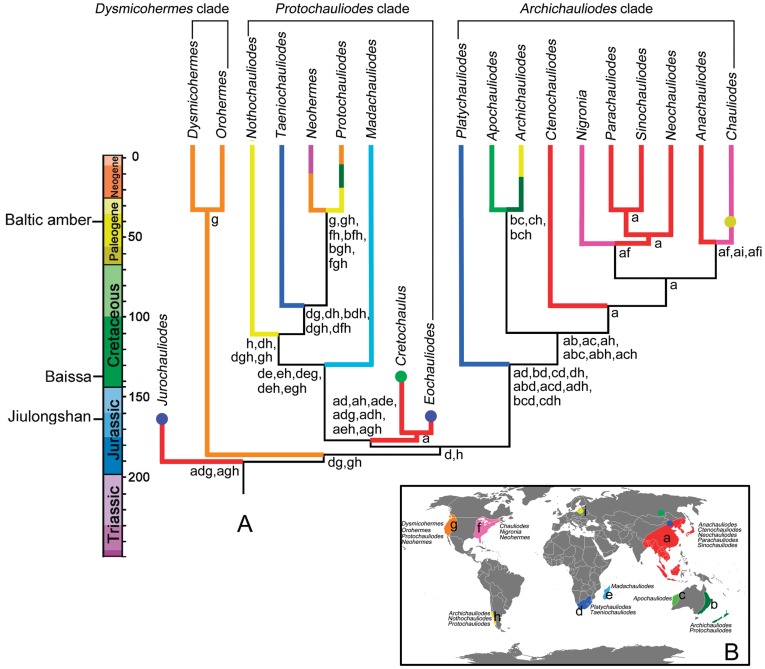
Ancestral distribution reconstruction of fishflies under DIVA analysis along one of the most parsimonious trees. A, Cladogram with geologic periods calibrated to a time scale in millions of years at left. Fossil localities of fishflies marked at far left. Circles indicate the fossil records. Color of branches corresponds to the distributed areas of fishfly genera shown in Fig. 4B, branches with mixed color indicate disjunctive distribution among different areas. B, Distribution map of world fishflies, circles indicate the localities of fossil fishflies. Areas are coded as follows: a, Asia; b, eastern Australia; c, western Australia, d, southern African continent; e, Madagascar; f, eastern North America; g, western North America; h, southern South America; i, Europe.

## Materials and Methods

### Specimens Examined and Terminology

The fossil specimens were examined using a Leica M165C dissecting microscope and illustrated with the aid of a drawing tube. Photos of all specimens were taken by Nikon D90 and Leica DFC500 digital cameras. All fossil specimens described herein are deposited in the Key Lab of Insect Evolution & Environmental Changes, Capital Normal University, Beijing (CNU). The specimens of extant Chauliodinae and Sialidae examined for the phylogenetic analysis are deposited in the Entomological Museum, China Agricultural University, Beijing (CAU); the National Science Museum, Tokyo (NSM); the National Museum of Natural History, Smithsonian Institution, Washington, DC (NMNH); the Natural History Museum, London (NHM); the Iziko South African Museum, Cape Town (SAMC); the Albany Museum, Grahamstown (AMGS), and the Australian Museum, Sydney (AMS). Terminology of wing venation follows Wootton [60].

### Nomenclatural Acts

The electronic version of this document does not represent a published work according to the International Code of Zoological Nomenclature (ICZN), and hence the nomenclatural acts contained in the electronic version are not available under that Code from the electronic edition. Therefore, a separate edition of this document was produced by a method that assures numerous identical and durable copies, and those copies were simultaneously obtainable (from the publication date noted on the first page of this article) for the purpose of providing a public and permanent scientific record, in accordance with Article 8.1 of the Code. The separate print-only edition is available on request from PLoS by sending a request to PLoS ONE, 1160 Battery Street Suite 100, San Francisco, CA 94111, USA along with a check for $10 (to cover printing and postage) payable to “Public Library of Science”. In addition, this published work and the nomenclatural acts it contains have been registered in ZooBank, the proposed online registration system for the ICZN. The ZooBank LSIDs (Life Science Identifiers) can be resolved and the associated information viewed through any standard web browser by appending the LSID to the prefix “http://zoobank.org/”. The LSID for this publication is: urn:lsid:zoobank.org:pub:14656DD2-028F-4030-B314-8962EA9CAD0E.

### Phylogenetic Analysis

In order to clarify taxonomic status of the new fossil fishfly taxa, a phylogenetic analysis based on morphological data was conducted including all known extinct and extant genera worldwide (Table S1). The morphological data were obtained mainly from the wing venation, adult genitalic structures, and larval morphology, which are available for most ingroup taxa (Figs. S2, S3, S4). The morphological character list and the data matrix are provided in the Table S2 and Text S1. Some character states are obtained from literatures [1], [4], [12]–[17], [22], [29], [30], [61]–[64]. We selected four genera as outgroup taxa, namely *Ororaphidia* Engel & Ren (Raphidioptera) [65], *Leptosialis* Esben-Petersen (Sialidae) [66], *Chloroniella* Esben-Petersen (Corydalinae) [67], and *Platyneuromus* van der Weele (Corydalinae) [68]. The outgroup selection is basing on the phylogenetic relationships among Neuropterida: Raphidioptera is considered to be the sister group of Megaloptera and Neuroptera, Sialidae traditionally represents the sister group of Corydalidae, and Corydalinae is the sister group of Chauliodinae [8].

The phylogenetic analysis was performed in PAUP* version 4.0b10 [69] by using a heuristic parsimony analysis, with 100 random stepwise additions of taxa (TBR branch swapping), characters unordered and of equal weight, MulTrees option in effect. Bootstrap values for clades were calculated in 1000 replicates using a general heuristic search. Bremer’s decay index was calculated with Autodecay version 4.0 [70] and PAUP* version 4.0b10. The unambiguous characters were mapped by MacClade version 4.0 [71].

### Biogeographical Analysis

Based on the geographical distribution patterns of fishflies [4], we categorized the distributions of the world fishflies into the following endemic areas: Asia (a), eastern Australia (b), western Australia (c); southern African continent (d); Madagascar (e); eastern North America (f); western North America (g); southern South America (h); Europe (i) (Fig. 4B).

We constructed the historical biogeography of Chauliodinae using a dispersal-vicariance (DIVA) optimization model [72] implemented in the programme RASP2.0 Beta [73]. The DIVA model acknowledges the need for some level of dispersal in explaining the occurrence of widespread ancestors, and the optimal ancestral reconstruction of the DIVA model is the one with the least cost, i.e. the most parsimonious. DIVA requires that the phylogenetic relationships among taxa are fully resolved, therefore we used one of the MP trees with completely binary topology for this analysis. The DIVA optimization was conducted with default settings except for the maximum number of areas in ancestral ranges constrained to three. The most widespread fishfly genera, *Protochauliodes*, are distributed in three of the defined area units (eastern Australia, southern South America, and western North America). The constraint on ancestral ranges reflects the assumption that the ranges of ancestral begonias were similar to those of their extant descendants [74].

## Supporting Information

Figure S1 **Strict consensus tree from the phylogenetic analysis.**
(TIF)

Figure S2 **Habitus of fishfly species, with selected characters on wings.** A, *Dysmicohermes disjunctus* (Walker); B, *Orohermes crepusculus* (Chandler); C, *Madachauliodes torrentialis* Paulian; D, *Platychauliodes pusillus* (McLachlan); E, *Apochauliodes cervulus* Theischinger; F, *Archichauliodes neoguttiferus* Theischinger. Scale bars: 5.0 mm.
(TIF)

Figure S3 **Habitus of fishfly species, with selected characters on wings.** A, *Taeniochauliodes esbenpeterseni* Kimmins; B, *Nothochauliodes penai* Flint; C, *Protochauliodes kirramae* Theischinger; D, *Neohermes californicus* (Walker). Scale bars: 5.0 mm.
(TIF)

Figure S4 **Habitus of fishfly species, with selected characters on wings.** A, *Chauliodes rastricornis* Rambur; B, *Anachauliodes tonkinicus* Kimmins; C, *Ctenochauliodes elongatus* Liu & Yang; D, *Nigronia serricornis* (Say); E, *Neochauliodes confusus* Liu, Hayashi & Yang; F, *Parachauliodes continentalis* van der Weele; G, *Sinochauliodes squalidus* Liu & Yang. Scale bars: 5.0 mm.
(TIF)

Table S1 **Taxa sampling in the present cladistic analysis and their geographical distributions.**
(DOC)

Table S2 **Data matrix of characters and character states.**
(DOC)

Text S1 **Character states coded for Chauliodinae.**
(DOC)
