# Supplementary figures and images for: Early Evolution and Historical Biogeography of Fishflies (Megaloptera: Chauliodinae): Implications from a Phylogeny Combining Fossil and Extant Taxa
Source: PLoS One. 2012 Jul 6;7(7):e40345. doi: 10.1371/journal.pone.0040345 (PMC3391272; doi:10.1371/journal.pone.0040345)

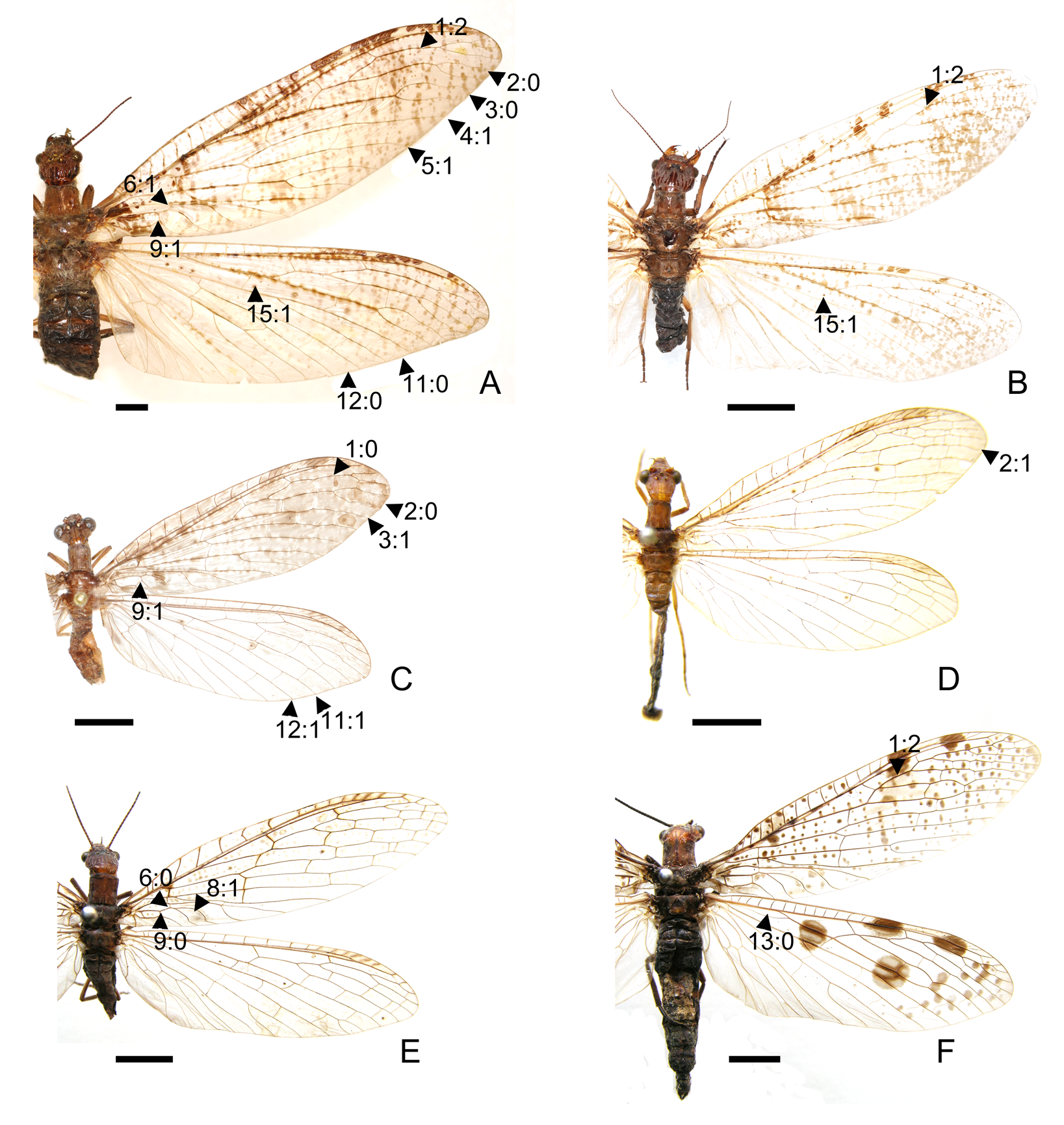

Supplement: Figure S2 — Habitus of fishfly species, with selected characters on wings. A, Dysmicohermes disjunctus (Walker); B, Orohermes crepusculus (Chandler); C, Madachauliodes torrentialis Paulian; D, Platychauliodes pusillus (McLachlan); E, Apochauliodes cervulus Theischinger; F, Archichauliodes neoguttiferus Theischinger. Scale bars: 5.0 mm. (TIF) [file pone.0040345.s002.tif]

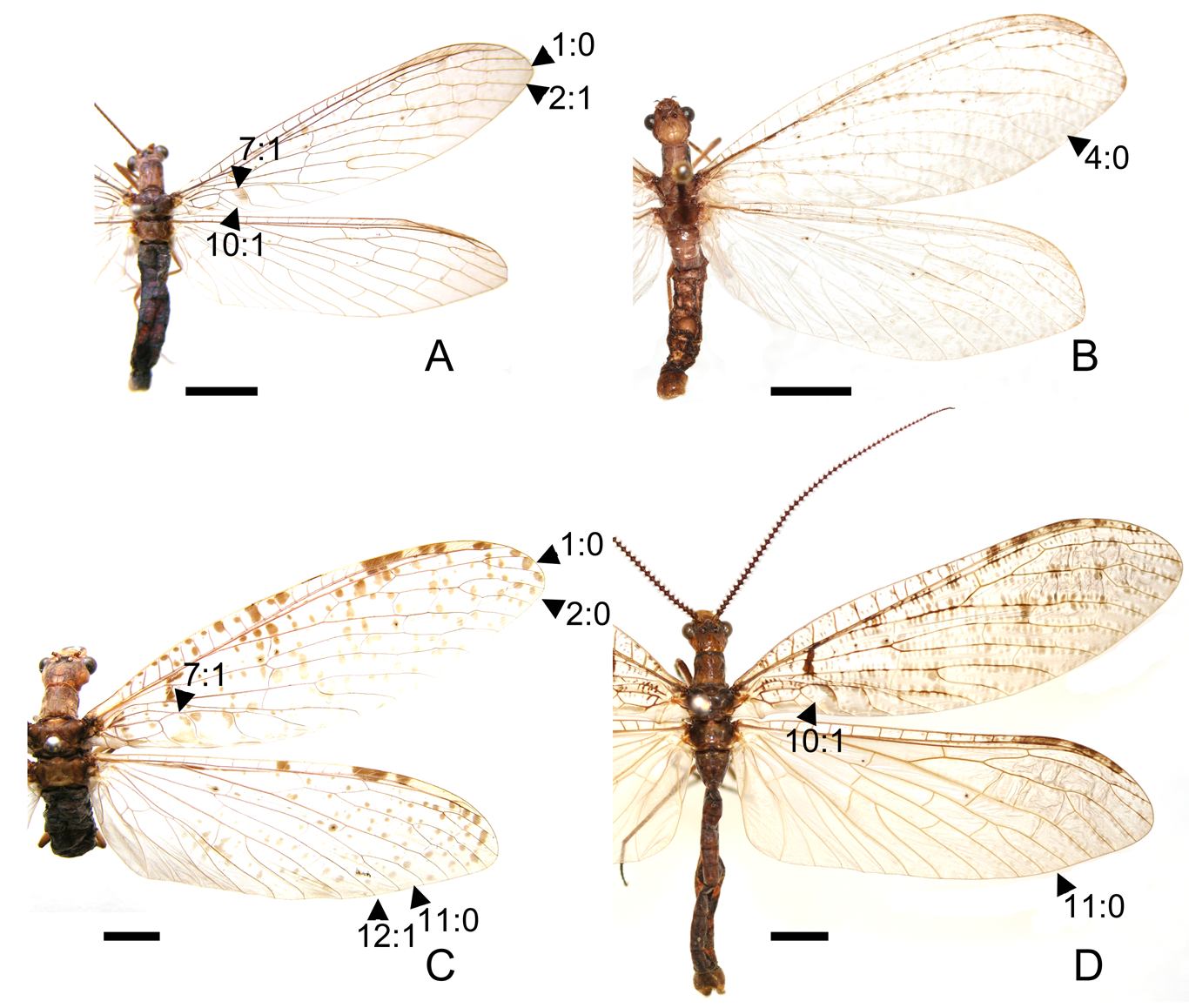

Supplement: Figure S3 — Habitus of fishfly species, with selected characters on wings. A, Taeniochauliodes esbenpeterseni Kimmins; B, Nothochauliodes penai Flint; C, Protochauliodes kirramae Theischinger; D, Neohermes californicus (Walker). Scale bars: 5.0 mm. (TIF) [file pone.0040345.s003.tif]

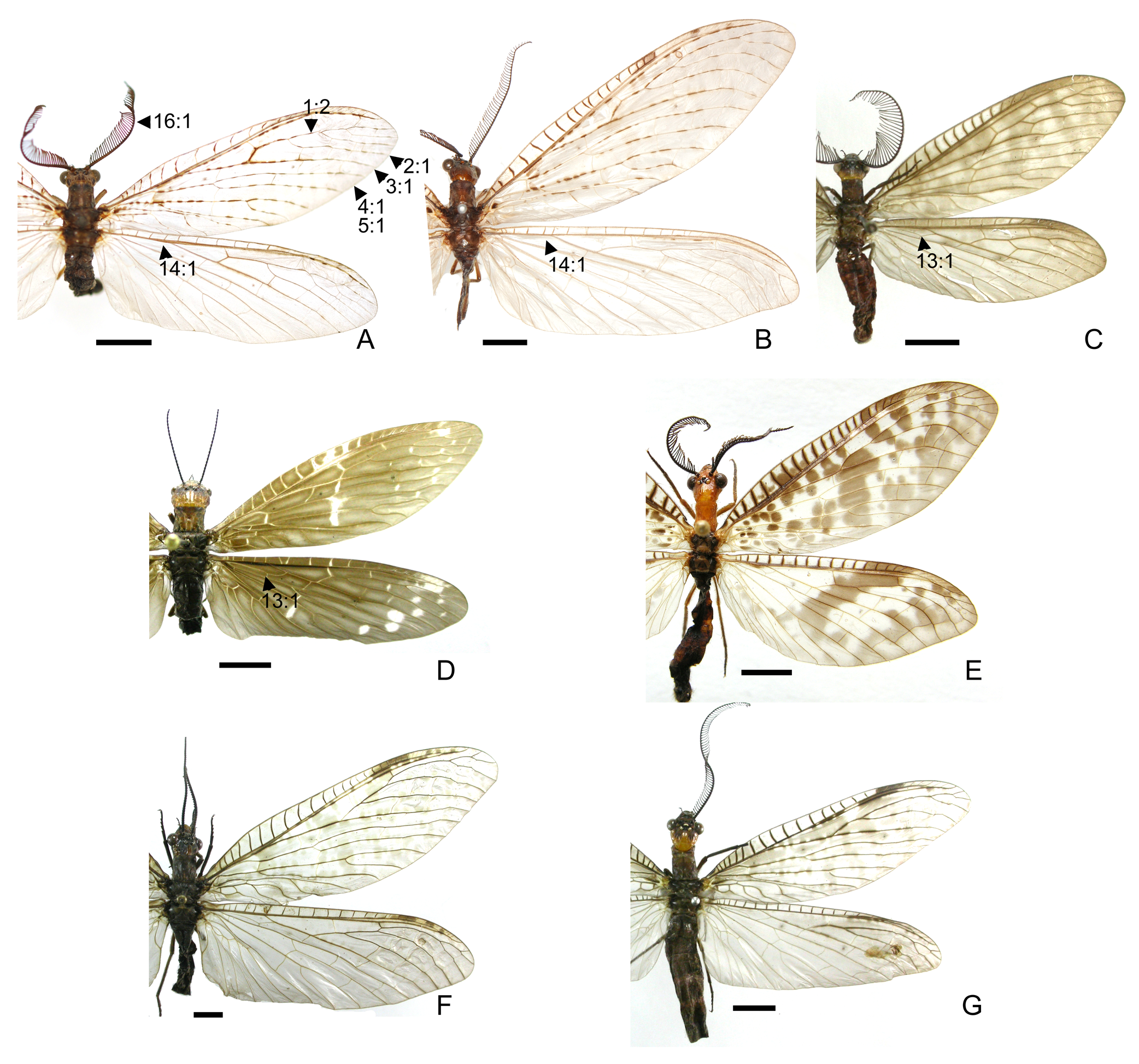

Supplement: Figure S4 — Habitus of fishfly species, with selected characters on wings. A, Chauliodes rastricornis Rambur; B, Anachauliodes tonkinicus Kimmins; C, Ctenochauliodes elongatus Liu & Yang; D, Nigronia serricornis (Say); E, Neochauliodes confusus Liu, Hayashi & Yang; F, Parachauliodes continentalis van der Weele; G, Sinochauliodes squalidus Liu & Yang. Scale bars: 5.0 mm. (TIF) [file pone.0040345.s004.tif]
