# Supplementary material for: Early Evolution and Historical Biogeography of Fishflies (Megaloptera: Chauliodinae): Implications from a Phylogeny Combining Fossil and Extant Taxa
Source: PLoS One. 2012 Jul 6;7(7):e40345. doi: 10.1371/journal.pone.0040345 (PMC3391272; doi:10.1371/journal.pone.0040345)
Supplement: Table S1 — Taxa sampling in the present cladistic analysis and their geographical distributions. (DOC) [file pone.0040345.s005.doc]

**Table S1 Taxa sampling in the present cladistic analysis and their geographical distributions.**

| Taxa | Distribution |
| --- | --- |
| **RAPHIDIOPTERA**  **†*Ororaphidia* Engel & Ren, 2008**  **†***Ororaphidia megalocephala* Engel & Ren, 2008  **MEGALOPTERA**  **SIALIDAE**  ***Leptosialis* Esben-Petersen, 1924**  *Leptosialis* sp.  **CORYDALIDAE**  **CORYDALINAE**  ***Chloroniella* Esben-Petersen, 1924**  *Chloroniella peringueyi* Esben-Petersen, 1924  ***Platyneuromus* van der Weele, 1909**  *Platyneuromus soror* (Hagen, 1861)  **CHAULIODINAE**  ***Anachauliodes* Kimmins, 1954**  *Anachauliodes tonkinicus* Kimmins, 1954  ***Apochauliodes* Theischinger, 1983**  *Apochauliodes cervulus* Theischinger, 1983  ***Archichauliodes* van der Weele, 1909**  *Archichauliodes uncinatus* Kimmins, 1954  ***Chauliodes* Latreille, 1802**  *Chauliodes rastricornis* Rambur, 1842  **†*Cretochaulus* Ponomarenko, 1976**  **†***Cretochaulus lacustris* Ponomarenko, 1976  ***Ctenochauliodes* van der Weele, 1909**  *Ctenochauliodes yangi* Liu & Yang, 2006  ***Dysmicohermes* Munroe, 1953**  *Dysmicohermes disjunctus* (Walker, 1866)  **†*Eochauliodes* gen. n.**  **†*Eochauliodes striolatus* sp. n.**  **†*Jurochauliodes* Wang & Zhang, 2010**  **†*Jurochauliodes ponomarenkoi* Wang & Zhang, 2010**  ***Madachauliodes*** **Paulian, 1951**  *Madachauliodes torrentialis* Paulian, 1951  ***Neochauliodes* van der Weele, 1909**  *Neochauliodes confusus* Liu, Hayashi & Yang, 2010  ***Neohermes* Banks, 1908**  *Neohermes californicus*  ***Nigronia* Banks, 1908**  *Nigronia fasciatus* (Walker, 1853)  ***Nothochauliodes* Flint, 1983**  *Nothochauliodes penai* Flint, 1983  ***Orohermes* Evans, 1984**  *Orohermes crepusculus* (Chandler, 1954)  ***Parachauliodes* van der Weele, 1909**  *Parachauliodes japonicus* (McLachlan, 1867)  ***Platychauliodes* Esben-Petersen, 1924**  *Platychauliodes capensis* Barnard, 1931  ***Protochauliodes* van der Weele, 1909**  *Protochauliodes biconicus* Flint, 1973  ***Sinochauliodes* Liu & Yang, 2006**  *Sinochauliodes squalidus* Liu & Yang, 2006  ***Taeniochauliodes* Esben-Petersen, 1924**  *Taeniochauliodes esbenpeterseni* Kimmins, 1930 | Middle Jurassic of China  South Africa  South Africa  Central America  China, Vietnam  Australia  Australia, Chile, New Zealand  Canada (Eastern), USA (Eastern)  Early Cretaceous of Russia  China, India, Vietnam  Canada (Western), USA (Western)  Middle Jurassic of China  Middle Jurassic of China  Madagascar  Widespread in East, South East and South Asia  Mexico, USA  USA (Eastern)  Chile  USA (Western)  Japan, Korea, Taiwan  South Africa  Australia, Chile, Canada (Western), USA (Western)  China  South Africa |
