# Supplementary material for: Early Evolution and Historical Biogeography of Fishflies (Megaloptera: Chauliodinae): Implications from a Phylogeny Combining Fossil and Extant Taxa
Source: PLoS One. 2012 Jul 6;7(7):e40345. doi: 10.1371/journal.pone.0040345 (PMC3391272; doi:10.1371/journal.pone.0040345)
Supplement: Table S2 — Data matrix of characters and character states. (DOC) [file pone.0040345.s006.doc]

**Table S2** **Data matrix of characters and character states.**

|  | 0 | 0 | 0 | 0 | 0 | 0 | 0 | 0 | 0 | 1 | 1 | 1 | 1 | 1 | 1 | 1 | 1 | 1 | 1 | 2 | 2 | 2 | 2 | 2 | 2 | 2 | 2 | 2 | 2 | 3 | 3 | 3 | 3 | 3 | 3 | 3 | 3 | 3 | 3 | 4 | 4 |
| --- | --- | --- | --- | --- | --- | --- | --- | --- | --- | --- | --- | --- | --- | --- | --- | --- | --- | --- | --- | --- | --- | --- | --- | --- | --- | --- | --- | --- | --- | --- | --- | --- | --- | --- | --- | --- | --- | --- | --- | --- | --- |
|  | 1 | 2 | 3 | 4 | 5 | 6 | 7 | 8 | 9 | 0 | 1 | 2 | 3 | 4 | 5 | 6 | 7 | 8 | 9 | 0 | 1 | 2 | 3 | 4 | 5 | 6 | 7 | 8 | 9 | 0 | 1 | 2 | 3 | 4 | 5 | 6 | 7 | 8 | 9 | 0 | 1 |
| *Ororaphidia*  *Leptosialis*  *Chloroniella*  *Platyneuromus*  *Anachauliodes*  *Apochauliodes*  *Archichauliodes*  *Chauliodes*  *Cretochaulus*  *Ctenochauliodes*  *Dysmicohermes*  *Eochauliodes*  *Jurochauliodes*  *Madachauliodes*  *Neochauliodes*  *Neohermes*  *Nigronia*  *Nothochauliodes*  *Orohermes*  *Parachauliodes*  *Platychauliodes*  *Protochauliodes*  *Sinochauliodes*  *Taeniochauliodes* | 0  1  2  2  2  2  2  2  0  2  2  0  2  0  2  0  2  0  2  2  2  0  2  0 | 1  1  1  1  1  1  1  1  0  1  0  0  0  0  1  0  1  1  0  1  1  0  1  1 | 0  0  1  1  1  1  1  1  1  1  0  0  0  1  1  1  1  1  0  1  1  1  1  1 | 0  0  0  0  1  1  1  1  0  1  1  0  0  1  1  1  1  0  1  1  1  1  1  1 | 0  0  1  0  1  1  1  1  1  1  1  1  0  1  1  1  1  1  1  1  1  1  1  1 | 1  0  1  1  1  0  0  1  ?  1  1  0  0  1  0  1  0  1  1  0  1  1  0  1 | 0  0  0  0  0  0  0  0  ?  0  0  0  0  0  0  1  0  1  0  0  0  1  0  1 | 0  0  0  0  0  1  1  0  ?  1  0  1  0  1  0  1  1  1  0  1  1  1  1  1 | ?  0  1  1  0  0  0  0  ?  0  1  ?  ?  1  0  1  0  1  1  0  0  1  0  1 | 0  0  0  0  0  0  0  0  0  0  0  0  0  1  0  1  0  1  0  0  0  1  0  1 | 0  0  0  0  1  1  1  1  0  1  0  0  0  1  1  0  1  0  0  1  1  0  1  1 | 0  0  1  0  1  1  1  1  1  1  0  1  0  1  1  1  1  1  1  1  1  1  1  1 | 0  0  0  0  0  0  0  0  0  1  0  0  0  0  1  0  1  0  0  1  0  0  1  0 | 0  0  0  0  1  0  0  1  0  0  0  0  0  0  0  0  0  0  0  0  0  0  0  0 | ?  0  0  0  0  0  0  0  0  0  1  0  ?  0  0  0  0  0  1  0  0  0  0  0 | 0  0  0  0  1  0  0  1  0  1  0  0  ?  0  1  0  1  0  0  1  0  0  1  0 | ?  0  0  0  ?  0  0  0  0  ?  1  0  1  0  0  0  0  ?  1  0  0  0  ?  0 | 0  0  0  0  1  1  1  1  1  1  1  1  1  1  1  1  1  1  1  1  1  1  1  1 | 0  0  1  1  1  1  1  1  1  1  1  1  1  1  1  1  1  1  1  1  1  1  1  1 | 0  0  1  1  1  1  1  1  1  1  1  1  1  1  1  1  1  1  1  1  1  1  1  1 | 0  0  0  0  ?  1  1  1  0  ?  0  0  0  0  1  0  1  ?  0  1  1  0  ?  0 | ?  0  1  1  0  0  0  0  0  0  0  0  0  0  0  0  0  0  0  0  0  0  0  0 | ?  0  0  0  1  0  0  1  ?  0  0  ?  ?  0  1  0  1  0  0  1  0  0  1  0 | ?  0  0  0  0  0  0  0  ?  0  0  ?  ?  0  0  1  0  1  0  0  0  1  0  1 | ?  0  0  0  0  0  0  0  ?  0  0  ?  ?  0  0  1  0  0  0  0  0  1  0  1 | ?  0  0  0  0  1  1  0  ?  0  0  ?  ?  0  1  0  1  0  0  1  0  0  1  0 | ?  0  0  0  1  1  1  1  ?  1  1  ?  ?  1  1  1  1  1  1  1  1  1  1  1 | ?  0  0  0  1  1  1  1  ?  1  0  ?  ?  0  1  0  1  0  0  1  1  0  1  0 | ?  0  0  0  ?  ?  ?  ?  ?  ?  0  ?  ?  1  ?  1  ?  1  0  ?  ?  1  ?  1 | ?  0  0  0  0  0  0  0  ?  0  0  ?  ?  0  1  0  1  0  0  1  0  0  1  0 | ?  0  0  0  1  0  0  1  ?  0  0  ?  ?  0  0  0  0  0  0  0  0  0  0  0 | ?  0  0  0  0  0  0  0  ?  0  1  ?  ?  0  0  0  0  0  1  0  0  0  0  0 | ?  0  0  0  0  0  0  0  ?  0  0  ?  ?  0  1  0  0  0  0  1  0  0  1  0 | ?  0  0  1  0  0  0  0  ?  0  0  ?  ?  0  0  1  0  0  0  0  0  1  0  1 | ?  0  1  1  0  0  0  0  ?  0  0  ?  ?  0  0  0  0  0  0  0  0  0  0  0 | ?  0  0  0  0  0  0  0  ?  0  0  ?  ?  0  0  0  0  0  0  1  0  0  1  0 | ?  ?  0  0  1  0  0  1  ?  0  0  ?  ?  1  1  1  1  0  0  1  0  1  1  1 | ?  0  0  0  0  0  0  0  ?  0  0  ?  ?  1  0  1  0  1  0  0  0  1  0  1 | ?  0  0  0  1  0  0  1  ?  0  0  ?  ?  0  1  0  1  ?  0  1  0  0  1  0 | ?  0  0  0  1  1  1  1  ?  1  0  ?  ?  0  1  0  1  ?  0  1  1  0  1  0 | ?  0  1  1  0  0  0  0  ?  0  1  ?  ?  0  0  0  0  ?  1  0  0  0  0  0 |
