# Supplementary material for: Early Evolution and Historical Biogeography of Fishflies (Megaloptera: Chauliodinae): Implications from a Phylogeny Combining Fossil and Extant Taxa
Source: PLoS One. 2012 Jul 6;7(7):e40345. doi: 10.1371/journal.pone.0040345 (PMC3391272; doi:10.1371/journal.pone.0040345)
Supplement: Text S1 — Character states coded for Chauliodinae. (DOC) [file pone.0040345.s007.doc]

1. Forewing with anterior branch of Rs: (0) bifurcated (Fig. S2C); (1) simple; (2) more than two branches (Fig. S2A). The Rs of the forewing is proximally bifurcated into two main branches and each main branch has different states of additional branching ranging simple to over two branches. In various primitive groups of holometabolous insects, such as Miomoptera (Kukalová-Peck, 1991) and Raphidioptera (see the outgroup species *O. megalocephala*), the anterior main branch of Rs possesses only two subbranches, which is probably plesiomorphic.
2. Forewing with posterior branch of Rs: (0) bifurcated (Fig. 5A); (1) simple (Fig. S2D). The posterior main branch of Rs of the forewing is considered to be similarly evolved as the anterior main branch of Rs from a bifurcated fork to a simple vein in Chauliodinae.
3. Forewing with MA: (0) bifurcated (Fig. S2A); (1) simple (Fig. S2C). The basically bifurcated MA of the forewing is present in both outgroup taxa and many other stem groups of holometabolous insects, and it is considered to be plesiomorphic. Only four ingroup taxa, *Jurochauliodes*, *Eochauliodes*, *Dysmicohermes*, and *Orohermes*, have bifurcated MA in the forewings.
4. Forewing with anterior branch of MP: (0) having over two subbranches (Fig. 1G); (1) simple (Fig. S2A). The MP of the forewing is proximally bifurcated into two main branches, and the anterior branch plesiomorphically has two or more subbranches in the outgroup taxa and many other basal groups of the holometabolous insects, such as Neuroptera, Mecoptera, and Amphiesmenoptera (Kukalová-Peck, 1991).
5. Forewing with posterior branch of MP: (0) bifurcated (Fig. 2B); (1) simple (Fig. S2A). The posterior branch of MP of the forewing is similarly evolved as the anterior branch of MP.
6. Forewing with 1A and 2A connected: (0) at stem of 2A (Fig. S2E); (1) at anterior branch of 2A (Fig. S2C). The 1A and 2A is connected by a crossvein or a fusion in the forewing of all Megaloptera species, however, the connecting point on 2A is variable among different genera. The plesiomorphic state is that the connection is present at the stem of 2A.
7. Forewing with 1A and 2A: (0) attached by a crossvein (Fig. S2E); (1) attached by a fusion for a short distance (Fig. S3A). The anterior branch of 2A is fused for a short distance with the stem of 1A in the forewings of *Protochauliodes*, *Neohermes*, *Taeniochauliodes*, and *Nothochauliodes*, and this state is considered to be apomorphic.
8. Forewing with anterior branch of 2A: (0) feebly curved (Fig. 2D); (1) strongly curved (Fig. S2E). The anterior branch of 2A on the forewing is generally extending without distinct curve in Megaloptera, however, it is strongly curved into a sinuate vein in many genera of Chauliodinae.
9. Forewing with 2A and 3A connected: (0) at stem of 2A (Fig. S2E); (1) at posterior branch of 2A (Fig. S2A). Similar to character 6, the 2A and 3A is also connected by a crossvein in the forewing of all Megaloptera species, however, the connecting point on 2A is variable among different genera. The plesiomorphic state is that the connection is present at the stem of 2A.
10. Forewing with posterior branch of 2A: (0) curved (Fig. S2E); (1) straightly extending (Fig. S3A). The posterior branch of 2A of the forewing is generally curved to some degree in most fishfly genera and the outgroups species, however, it is straightly extending as an attenuate veinlet, which is apomorphic, in *Protochauliodes*, *Neohermes*, *Taeniochauliodes*, *Nothochauliodes*, and *Madachauliodes*.
11. Hindwing with anterior branch of MP: (0) bifurcated (Fig. S2A); (1) simple (Fig. S2C). Similar to character 4, the simple anterior branch of MP of the forewing is considered to be apomorphic and present in most ingroup taxa except *Jurochauliodes*, *Eochauliodes*, *Cretochaulus*, *Protochauliodes*, *Nothochauliodes*, *Neohermes*, *Dysmicohermes*, and *Orohermes*.
12. Hindwing with posterior branch of MP: (0) bifurcated (Fig. S2A); (1) simple (Fig. S2C). Similar to character 5, the simple posterior branch of MP of the forewing is considered to be apomorphic and present in most ingroup taxa except *Jurochauliodes* and *Dysmicohermes*.
13. Hindwing with base of MA: (0) simple (Fig. S2F); (1) bifurcated into an additional veinlet (Fig. S4C). The base of MA is generally visible as an independent oblique vein, interpreted as basal r-m crossvein by Kimmins (1954) and Liu and Yang (2006), connecting bases of Rs and MP in the hindwing. In *Ctenochauliodes*, *Neochauliodes*, *Nigronia*, *Parachauliodes*, and *Sinochauliodes* it reconnects to MP by an additional short branch or a short fusion, which is considered to be apomorphic.
14. Hindwing with base of MA: (0) present; (1) absent (Fig. S4A). The base of MA of the hindwing is absent in *Chauliodes* and *Anachauliodes* (at least absent in males of both genera), which is another apomorphic state of this vein.
15. Hindwing: (0) with two nygmata; (1) with more than five nygmata (Fig. S2A). The presence of nygmata is traditionally considered to be a plesiomorphic trait present in various relict groups of Neuropterida. All extant fishfly genera and the extinct *Eochauliodes* and *Cretochaulus* have several nygmata on the wings, and in most of these genera there are three nygmata on the forewing and two nygmata on the hindwing. However, in *Dysmicohermes* and *Orohermes* there are more than five nygmata in both wings, which is probably a derived apomorphic state.
16. Male antenna: (0) filiform or moniliform; (1) pectinate or subserrate (Fig. S4A). The antennal morphology is an important diagnostic character to distinguish some fishfly genera. The filiform or moniliform male antenna is considered to be plesiomorphic (Liu and Yang, 2006), while the subserrate or pectinate male antenna is apomorphic and shared by all extant Asian genera and two Nearctic genera (*Chauliodes* and *Nigronia*).
17. Larva with head: (0) as long as wide; (1) wider than long. The larval head has rather conservative shape among three major groups of Megaloptera (i.e. Sialidae, Chauliodinae, and Corydalidae), with length of head nearly equal to its width (Contreras-Ramos, 1998; Theischinger, 1999). However, in *Jurochauliodes*, *Dymicohermes*, and *Orohermes* the larval head is slightly shortened and wider than long (Evans, 1972; Wang and Zhang, 2010), which is considered to be apomorphic.
18. Larva with eighth abdominal spiracles: (0) normal; (1) specialized. The abdominal spiracles open on lateral sides of tergites 1-8 in Corydalinae with equal size among different segments, however, in Chauliodinae the abdominal spiracles on the eighth segment are specialized either as a pair of enlarged spiracles or protruding into a pair of spiracle tubes. The specialized eighth abdominal spiracles of the larva are considered to be a synapomorphic character of Chauliodinae.
19. Larva with a pair of prolegs on ninth abdominal segment: (0) absent; (1) present. All Corydalidae larvae possess a pair of prolegs on the ninth abdominal segment, and here it supports the grouping of the ingroup taxa.
20. Larva with a pair of lateral gills on eighth abdominal segment: (0) absent; (1) present. All Corydalidae larvae possess a pair of lateral gills on the eightth abdominal segment, and here it supports the grouping of the ingroup taxa.
21. Larva with eighth abdominal spiracles: (0) not protruding; (1) protruding into a pair of tubes. As the comment on character 18, the eighth abdominal spiracles of the fishfly larvae are specialized, and here the protrusion of these spiracles as a pair of tubes are a highly derived state, which is present in *Platychauliodes*, *Archichauliodes*, *Apochauliodes*, *Nigronia*, *Chauliodes*, *Neochauliodes*, and *Parachauliodes*.
22. Larva with abdominal ventral tufts: (0) absent; (1) present. The presence of abdominal ventral tufts is apomorphic for Corydalinae.
23. Male ninth sternum: (0) nearly as long as ninth tergum; (1) strongly shortened. The ninth sternum in males of *Anachauliodes*, *Chauliodes*, *Neochauliodes*, *Nigronia*, *Parachauliodes*, and *Sinochauliodes* strongly shortened into a small plate, which is considered to be apomorphic.
24. Male ninth sternum: (0) subtrapezoidal; (1) subquadrate. The male ninth sternum in many fishfly genera is somewhat narrowed toward apex, forming a subtrapezoidal plate, while in *Nothochauliodes*, *Taeniochauliodes*, *Protochauliodes*, and *Neohermes* it is broadly subquadrate.
25. Male ninth sternum: (0) posteriorly truncate or feebly convex; (1) posteriorly prominent medially. Most fishfly genera possess the male sternum with truncate or feebly convex posterior margin, however, in *Taeniochauliodes*, *Protochauliodes*, and *Neohermes* the male ninth sternum have the posterior margin prominent medially.
26. Male ninth sternum: (0) posteriorly without membranous lobe; (1) posteriorly with a membranous lobe. In *Archichauliodes*, *Apochauliodes*, *Ctenochauliodes*, *Neochauliodes*, *Nigronia*, *Parachauliodes*, and *Sinochauliodes* there is a membranous lobe protruding from the posterior margin of the male ninth sternum, which is considered to apomorphic.
27. Male ninth gonocoxite: (0) well developed; (1) degenerated. The degenerated male ninth gonocoxite is an important diagnosis and probably a synapomorphic character for fishflies, although we cannot examine this character in the fossil genera.
28. Male ninth gonocoxite: (0) present; (1) absent. Although the male ninth gonocoxite in fishflies is degenerated, it remains as a small setose sclerite in some genera, but is completely absent in *Platychauliodes*, *Archichauliodes*, *Apochauliodes*, *Ctenochauliodes*, *Anachauliodes*, *Chauliodes*, *Neochauliodes*, *Nigronia*, *Parachauliodes*, and *Sinochauliodes*.
29. Male ninth gonocoxite: (0) not fused with tenth gonocoxite; (1) fused with tenth gonocoxite. In *Madachauliodes*, *Taeniochauliodes*, *Protochauliodes*, and *Neohermes* the degenerated ninth gonocoxite is fused with the lateral arm of the tenth gonocoxite, which is considered to be apomorphic.
30. Male ectoproct: (0) distally without feebly sclerotized inflation; (1) distally with feebly sclerotized inflation. In *Neochauliodes*, *Nigronia*, *Parachauliodes*, and *Sinochauliodes* the male extoproct has the inner portion feebly sclerotized and inflated to some degree.
31. Male ectoproct: (0) distally without small sclerotized claw; (1) distally with small sclerotized claw. The male ectoproct distally possesses a small sclerotized claw, which supports the grouping of *Anachauliodes* and *Chauliodes*.
32. Male ectoproct: (0) laterally without membranous tubular process; (1) laterally with membranous tubular process. The male ectoproct laterally possesses a membranous tubular process, which is considered to a synapomorphic character for the monophyly of Megaloptera. However, this trait is absent in most genera of Megaloptera, and here we assume it could be a synapomorphic character for the sister pair of *Dysmicohermes* and *Orohermes*.
33. Male ectoproct: (0) without brush-like setae; (1) with brush-like setae. In *Neochauliodes*, *Parachauliodes*, and *Sinochauliodes* the male ectoproct bears brush-like setae distally, which is considered to be apomorphic.
34. Male ectoproct: (0) distally without angulate bifurcation; (1) distally with angulate bifurcation. The male ectoproct in *Taeniochauliodes*, *Protochauliodes*, and *Neohermes* distally bifurcated into an angulate lobe laterally and a globally convex inner lobe, which is considered to be apomorphic.
35. Male cercus: (0) fused with ectoproct; (1) separated from ectoproct. The male cercus is present between ninth tergum and ectoproct in Corydalinae, but fused with ectoproct in Chauliodinae.
36. Male tenth gonocoxite: (0) not enveloped by ninth tergum, laterally visible; (1) enveloped by ninth tergum, laterally invisible. The male tenth gonocoxite is generally not enveloped by ninth tergum, but it is laterally invisible by the enveloping of the ninth tergum in *Parachauliodes* and *Sinochauliodes*.
37. Male tenth gonocoxite: (0) paired or deeply bifurcated; (1) present as a single sclerite, sometimes with distal notch. The distinctly bifurcated median plate of the male tenth gonocoxite is considered to be plesiomorphic and shared by many genera of Chauliodinae, while in *Anachauliodes*, *Chauliodes*, *Nigronia*, *Neochauliodes*, *Parachauliodes*, and *Sinochauliodes* the male tenth gonocoxite has its median plate not bifurcated.
38. Male tenth gonocoxite: (0) proximally without membranous lobe; (1) proximally with membranous lobe. The male tenth gonocoxite is proximally connected with ninth tergum by lateral arms. Besides the lateral arms, in *Madachauliodes*, *Nothochauliodes*, *Taeniochauliodes*, *Protochauliodes*, and *Neohermes* the male tenth gonocoxite bears membramous lobe connecting between lateral arm and ninth sternum.
39. Female eighth gonocoxite in ventral view: (0) subquadrate or subtrapezoidal; (1) subtriangular. The female eighth gonocoxite is subquadrate or subtrapezoidal in many fishfly genera, except for *Anachauliodes*, *Chauliodes*, *Nigronia*, *Neochauliodes*, *Parachauliodes*, and *Sinochauliodes*, which have the eighth gonocoxite strongly produced posteriad into a subtriangular plate.
40. Female ninth gonocoxite with gonostylus: (0) present; (1) absent. In Corydalidae, the female gonostylus is placed at tip of the gonocoxite and plesiomorphically articulated to the gonocoxite. In most genera of Chauliodinae, the female gonostylus is absent, while it remains in *Dysmicohermes*, *Orohermes*, *Madachauliodes*, *Taeniochauliodes*, *Protochauliodes*, and *Neohermes*.
41. Female ectoproct: (0) not bilobed; (1) bilobed. The female ectoproct is laterally separated by cercus into a dorsal and a ventral lobe, which is present in Corydalinae and two fishfly genera (*Dysmicohermes* and *Orohermes*).
